# Evolution of Akaganeite in Rust Layers Formed on Steel Submitted to Wet/Dry Cyclic Tests

**DOI:** 10.3390/ma10111262

**Published:** 2017-11-02

**Authors:** Haigang Xiao, Wei Ye, Xiaoping Song, Yuantai Ma, Ying Li

**Affiliations:** 1 Laboratory for Corrosion and Protection, Institute of Metal Research, Chinese Academy of Sciences, Wencui Rd. 62, Shenyang 110016, China; hgxiao12s@imr.ac.cn (H.X.); ytma@imr.ac.cn (Y.M.); 2 University of Chinese Academy of Sciences, Beijing 100049, China; 3 Analysis and Testing Department (Testing Center), Institute of Metal Research, Chinese Academy of Sciences, Wenhua Rd. 72, Shenyang 110016, China; weiye@imr.ac.cn (W.Y.); xpsong@imr.ac.cn (X.S.)

**Keywords:** atmospheric corrosion, accelerated experiments, chloride, carbon steel

## Abstract

The evolution of akaganeite in rust layers strongly impacts the atmospheric corrosion behavior of steel during long-term exposure; however, the factors affecting the evolution of akaganeite and its mechanism of formation are vague. In this work, wet-dry cyclic corrosion tests were conducted to simulate long-term exposure. Quantitative X-ray diffraction analysis was employed to analyze variations in the relative amounts of akaganeite; scanning electron microscopy and electron probe microanalysis were used to study the migration of relevant elements in the rust layer, which could help elucidate the mechanism of akaganeite evolution. The results indicate that the fraction of akaganeite tends to decrease as the corrosion process proceeded, which is a result of the decrease in the amount of soluble chloride available and the ability of the thick rust layer to block the migration of relevant ions. This work also explores the location of akaganeite formation within the rust layer.

## 1. Introduction

Atmospheric corrosion products that form on carbon steel are composed mainly of ferric oxyhydroxides, such as goethite (α-FeOOH), lepidocrocite (γ-FeOOH), akaganeite (β-FeOOH) and magnetite (Fe_3_O_4_), and amorphous rust [1,2,3]. Since each phase has distinct properties, the composition of the rust layer imparts certain protective abilities and strongly influences the subsequent corrosion behavior of the steel [4,5,6]. With a prolonged exposure time, a rust layer will experience numerous wet-dry cycles, and the corrosion products will be converted into one another during these cycles. The protective ability of a rust layer varies as the number of wet-dry cycles changes. Among all of these constituents, the presence of akaganeite in rust is considered an important cause of the severe corrosion observed in marine atmospheres [7]. The mass fraction of akaganeite in rust is correlated with the corrosion loss of steel [5,7]. Consequently, understanding the evolution of akaganeite in the rust layer during repeated wet-dry cycles would help advance our ability to interpret and predict corrosion performance in specific environments, and it may also suggest a method for protection against corrosion.

During the process of corrosion via repeated wet-dry cycles, the formation and consumption of akaganeite (FeO_0.833_(OH)_1.167_Cl_0.16_ [8]) controls its evolution in the rust layer. The formation process of akaganeite is still under debate, but there seems to be unanimous agreement that it is a product of green rust [9,10], and a high content of chloride ions are required for its formation [11]. Additionally, akaganeite participates in cathodic reactions and is consumed because it can serve as a reductant [12]. The consumption of akaganeite accelerates the corrosion process [9]. Tanaka reported that akaganeite reacts with Fe in an aqueous environment in the absence of chloride to generate magnetite and goethite [13].

Although akaganeite is frequently detected in the rust layers formed on steel surfaces in marine atmospheric environments, there have been very few investigations into its relative abundance during long-term atmospheric corrosion. Nonetheless, both the presence of the early stage of akaganeite and the disappearance of akaganeite after long-term outdoor exposure have been reported. J. Wang [14] found that akaganeite disappeared gradually, between months 6 and 30, on weathering steel exposed to an atmospheric environment typical of a salt lake. S. J. Oh [15] investigated steel samples subjected to marine atmospheric environments, and no akaganeite was detected because it was consumed by electrochemical reactions [11]. Ma [16] detected akaganeite in samples exposed for 12 months, but it was absent in those same samples after an additional 12 months of exposure. They proposed that the akaganeite was converted to γ-Fe_2_O_3_. Based on the results of an energy dispersive spectroscopic analysis (EDS) analysis on the bottom of the rust layer, two effects may account for the absence of akaganeite. During the process of corrosion, the outer layer thickens, and it becomes more difficult for chloride ions to penetrate the outer layer. Meanwhile, the chloride ions deposited during the initial stage, are quickly consumed. Since the corrosion process during outdoor exposure tests is influenced simultaneously by various environmental parameters, it could not clarify what exactly controls the relative fraction of akaganeite [17]. Thus, laboratory experiments conducted under well-defined conditions are an ideal method to elucidate the evolution of akaganeite during atmospheric corrosion.

Since deposited salt and abundant chloride ions are required to form akaganeite [18,19], the local distribution and migration of these materials in the rust layer significantly influences the evolution of akaganeite. Thus, an investigation into the migration process of salt and chloride would shed light on the evolution of akaganeite and assist in understanding its mechanism. Generally, salt ionizes to Na^+^ and Cl^−^ when it is dissolved in an electrolyte solution during the wet stage, and it recrystallizes when its concentration exceeds the saturation point of the solution during evaporation in the drying process. The locations of the salt and chloride vary throughout the corrosion process. In many studies, EDS has been employed to investigate the distribution of chloride in the rust layer, and the aggregation of chloride in the interface between the rust layer and substrate has been proposed [19,20,21]. However, the migration processes of salt and chloride in the rust layer have not been clarified yet.

In this work, the long-term atmospheric corrosion process of steel was simulated by repeated wet-dry cycles in a laboratory setting. The corrosion tests were performed under two representative salt deposition conditions, to determine the factors that control the evolution of akaganeite. The components in the rust layer were mainly characterized by X-ray diffraction (XRD). A quantitative XRD analysis was performed using a program called MAUD (material analysis using diffraction). The microstructure of the cross-section was observed by scanning electron microscopy (SEM), and the change in the distribution of Fe, O, Cl and Na in the rust layer as the number of wet-dry cycles increased was monitored by electron probe microanalysis (EPMA). Finally, the factors controlling the evolution of akaganeite during atmospheric corrosion are proposed. 

## 2. Materials and Methods

### 2.1. Materials

Wet-dry cyclic corrosion tests were performed on carbon steel Q235. The composition (wt %) of Q235 was C 0.176, S 0.023, P 0.019, Mn 0.57, Si 0.233, and Cu 0.033, with Fe composing the remainder. The samples were cut into 10 mm × 15 mm × 5 mm pieces. They were successively ground on SiC paper to 1000 grit and then cleaned with ethanol ultrasonically, rinsed with distilled water, dried and stored in a moisture-free desiccator prior to use. The corrosion solution was prepared from analytically pure sodium chloride and distilled water.

### 2.2. Characterization of the Iron Rust Phase

During characterization of the rust phases, all the rust was scraped from the metallic substrates of the six samples using a razor blade. The tip angle of the razor blade was 45°, which aided in removal of rust present in the pits on the surface. Almost all the adherent rust was removed using the razor blade, which was confirm by XRD analysis of the steel surface after rust removal (not shown). The scraped rust was mixed and ground to form a powder that appeared homogeneous. XRD measurements were performed with a Rigaku-D/max 2500 PC diffractometer (Tokyo, Japan) equipped with a Cu X-ray tube. A current of 300 mA and a voltage of 50 kV were employed as the tube settings. The XRD data were collected over a 2θ range of 5° to 85° with a step size of 0.02°. The data were collected for 5 s at each step. The quantitative XRD analysis was carried out using MAUD (version: 2.33, Luca Lutterotti, University of Trento, Trento, Italy), and the diffraction pattern fitting method was based on the Rietveld method [22]. The quantitative analysis was not accomplished until a sig parameter was between 1–2 and simultaneously the Rw parameter was less than 15.

For the Fourier Transform infrared spectroscopy (FTIR) analysis, approximately 3 mg of rust powder was mixed with approximately 100 mg of pure anhydrous KBr and ground to a fine powder in a mortar with a pestle. The mixture was pressed into a transparent circular flake approximately 1 mm thick. A Nicolet Corporation Model magan-IR560 infrared spectrophotometer (Thermo Fisher, Waltham, MA, USA) was used to acquire the infrared (IR) spectra of the rust powder in the range of 400 to 2000 cm^−1^. The spectra were recorded with Omnic software at a resolution of 4 cm^−1^, with 64 scans and a gain of 1.

For SEM observations, the samples were encapsulated in a PVC (Polyvinyl chloride) pipe filled with epoxy resin. The ring samples were polished with 2000 # grade emery paper and further polished with terylene, using coal oil to avoid dissolving the chloride in the rust layer. The micrographs of the cross-sections of the rust layers and the contents of each element in the rusts were obtained using SEM (INSPECT F50, FEI, Hillsboro, OH, USA) and energy dispersive X-ray diffraction (EDX, X-Max, OXFORD Instruments, Oxford, UK). The distributions of several important elements in the rust layers were detected by a Shimadzu Model EPMA-1610 electron probe microanalyzer (Kyoto, Japan) at an acceleration voltage of 15 KV and a sample current of 0.08 μA.

### 2.3. The Wet-Dry Cyclic Corrosion Tests

To thoroughly assess the factors controlling the evolution of akaganeite in different salt deposition conditions in different environments, two sets of conditions were simulated, namely, constant salt deposition throughout the whole process and sufficient fresh salt deposition in each cycle.

Under the first set of conditions, to maintain a constant deposited salt concentration in the rust layer during the whole process, all the salt in the experiments was deposited on the metal surface in the first wet-dry cycle, and deionized water was employed to wet the surface in subsequent wet-dry cycles. In the first wet-dry cycle, the samples were wet with 0.1 mL of 0.3 mol/L NaCl solution and dried at 30 °C with a relative humidity (RH) of 60% for 12 h. In the following wet-dry cycles, samples were wet with deionized water and then put in the drying environment. After 14, 28, 56, 84, and 112 cycles, a group of samples (15 pieces) was collected and characterized. Throughout the whole process, none of the deposited salt was washed away, and the concentration of deposited salt in the rust layer was constant. Under these conditions, all the salt (11,688 mg/m^2^) originated from the salt solution in the first cycle. To clarity the effects of the amount of salt initially deposited on the evolution of akaganeite, simulated experiments with salt depositions of 1168.8 and 116,880 mg/m^2^ were also conducted for 14 and 112 cycles, and the rust layer formed on those samples was characterized as well. These conditions are referred to as COND 1 in this paper.

Under the second set of conditions, an adequate supply of fresh salt was added to each wet-dry cycle. The specimens were wet with 0.1 mL of 3 mol/L NaCl and dried at 30 °C with a RH of 60% for 12 h in each cycle. Under these conditions, the amount of fresh salt deposited in each cycle was 116,880 mg/m^2^. To prevent the excess salt from accumulating on the surface of the rust layer, the samples were washed with 100 mL of deionized water three times. The washing could remove most of the salt deposits from previous cycles. After 14, 28, 42 and 112 cycles, one group of samples was taken out and analyzed. Before the analysis, the samples were washed in the same way as before. These conditions are referred to as COND 2 in this paper.

## 3. Results and Discussion

### 3.1. The Variations in the Relative Amounts of Akaganeite during Repeated Wet-Dry Cycles under the Two Sets of Conditions

**Figure 1:**
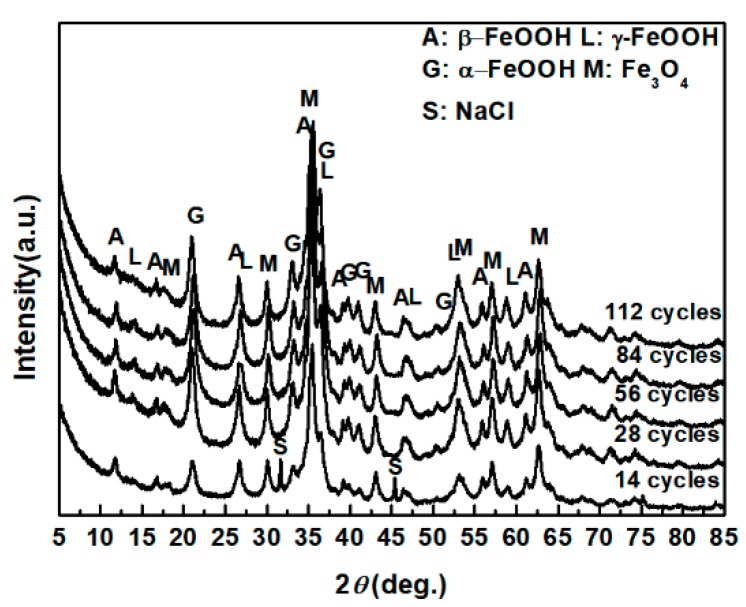
XRD patterns of the rust layer formed on the surfaces of samples after different numbers of cycles with constant salt deposition (11,688 mg/m^2^).

**Figure 2:**
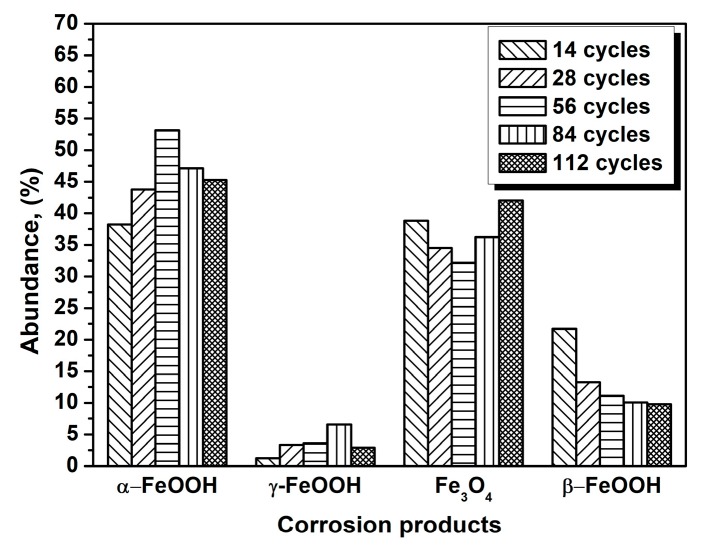
Composition in wt % of the rust layer formed on the surfaces of samples after different numbers of cycles with constant salt deposition (11,688 mg/m^2^); the data are shown in Table 1.

The evolution of akaganeite with 11,688 mg/m^2^ constant salt deposition was studied, and the XRD spectra of the corrosion products formed after 14, 28, 42, 86, and 112 cycles are shown in Figure 1. The rust contained goethite, lepidocrocite, akaganeite and magnetite regardless of the number of wet-dry cycles. Sodium chloride was detected in the rust powder when the sample experienced 14 cycles; however, it was absent from the subsequent rust samples. A quantitative XRD analysis was carried out using MAUD, and the results are summarized in Table 1. A histogram (Figure 2) was prepared from the data in Table 1 to better visualize the data. Under COND 1, with 11,688 mg/m^2^ constant salt deposition, the relative amount of akaganeite decreased as the number of wet-dry cycles increased.

**Figure 3:**
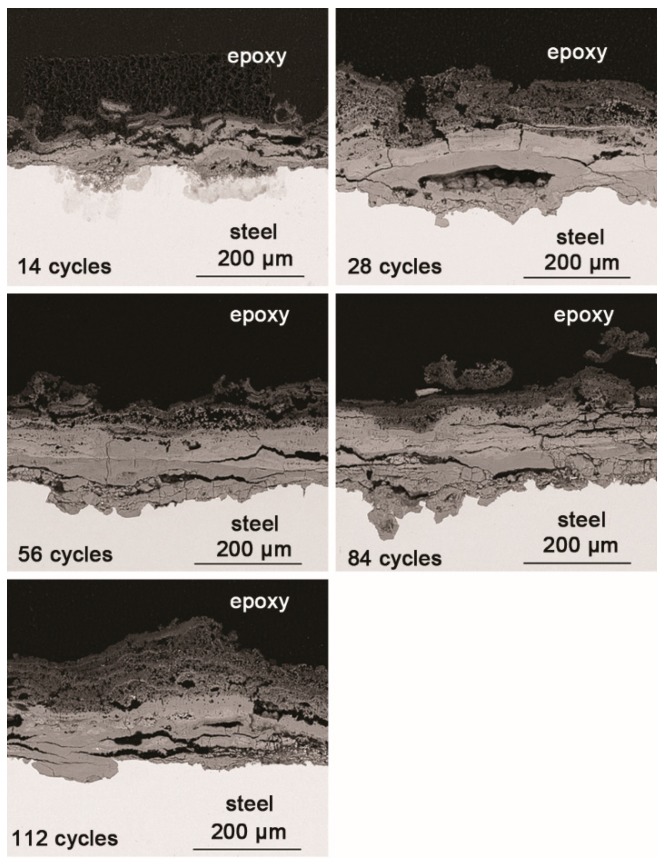
Cross-section SEM micrographs of steel specimens after different numbers of cycles with constant salt deposition (11,688 mg/m^2^).

SEM observations of the cross-sections of the rust layers after different cycles are shown in Figure 3. The corrosion reached deep into the metal in some locations and did not develop in other places; the thickness of the rust layer also varied by location. After 28 cycles, the rust layer was not obviously thickened. Since none of the corrosion products were washed away during the corrosion process, and all the corrosion products accumulated on the metal surface, the thickness of the rust layer could be indicative of how serious the corrosion was at a particular location. After 112 wet-dry cycles, the maximum thickness of the rust layer was approximately 200 μm. The outer layer of rust was loose, and the inner portion was relatively compact. Vertical and horizontal cracks were present in the rust layer.

**Figure 4:**
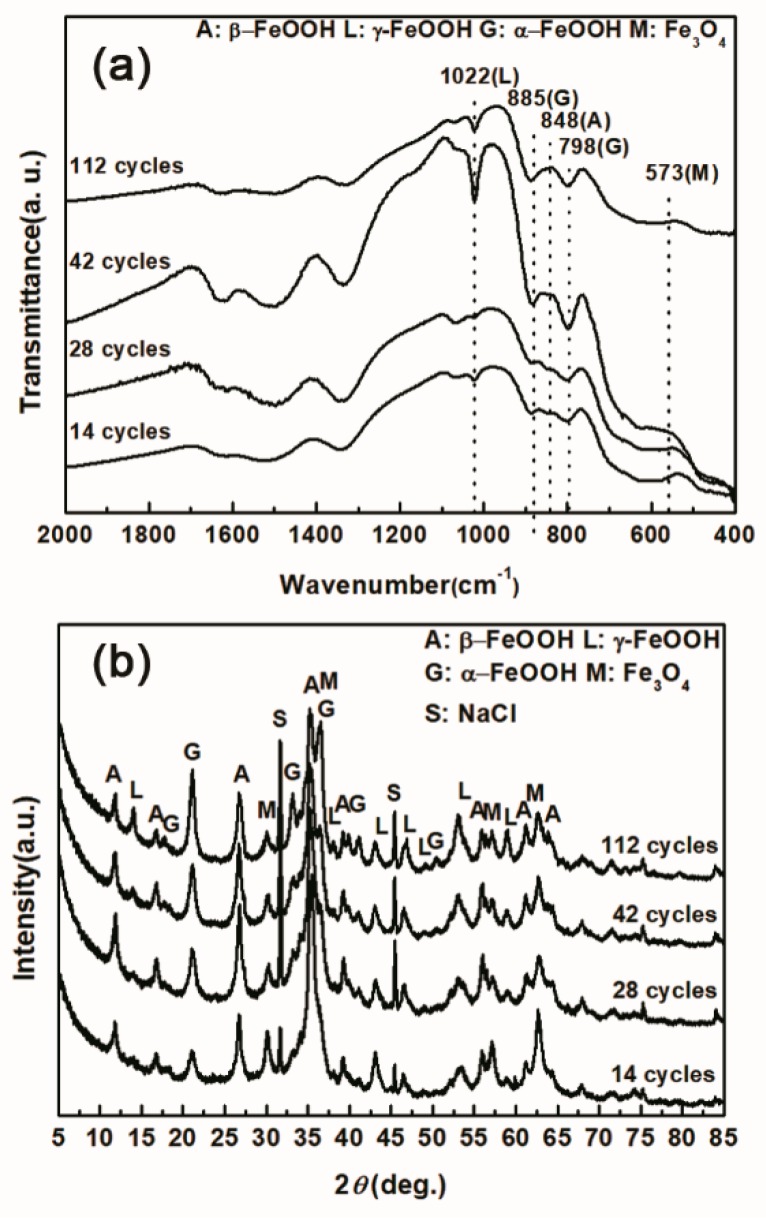
Infrared absorption spectrum (**a**) and X-ray diffraction (XRD) pattern (**b**) of the rust layer formed on the surfaces of samples after different numbers of cycles with sufficient fresh salt deposition in each cycle (>116,880 mg/m^2^).

**Figure 5:**
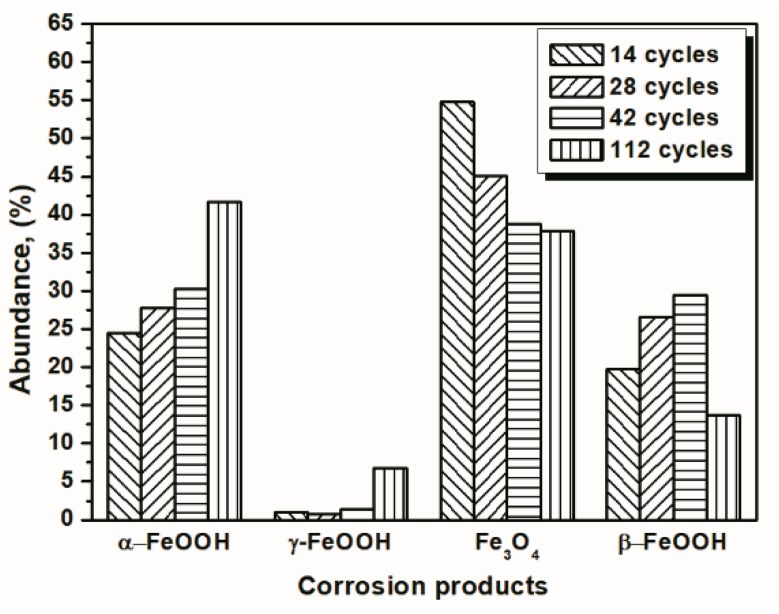
Composition in wt % of the rust layer formed on the surfaces of the samples after different numbers of cycles with sufficient fresh salt deposition in each cycle (>116,880 mg/m^2^); the data are shown in Table 2.

Under COND 2, the wet-dry cyclic corrosion test was conducted for 14, 28, 42 and 112 cycles. The rust layers were analyzed by FTIR and XRD, and those results are shown in Figure 4. In the FTIR spectra, the band at 1020 cm^−1^ is attributed to lepidocrocite, the band at 580 cm^−1^ is attributed to magnetite and the bands at 885 and 798 cm^−1^ are attributed to goethite [23]. The FTIR spectra demonstrated the presence of akaganeite through the characteristic bands at 848 cm^−1^ [24]. Both these techniques showed that lepidocrocite, goethite, akaganeite and magnetite formed regardless of the number of wet-dry cycles. NaCl crystals were detected after each cycle, which indicated that our washing procedure did not remove all the salt deposited, and the salt present in the rust layer during the corrosion process may have exceeded 11,6880 mg/m^2^. The results of the quantitative XRD analysis are listed in Table 2 and are presented as a bar graph in Figure 5. Between 14 cycles and 42 cycles, the fraction of akaganeite increased; however, its relative content decreased at 112 cycles.

**Figure 6:**
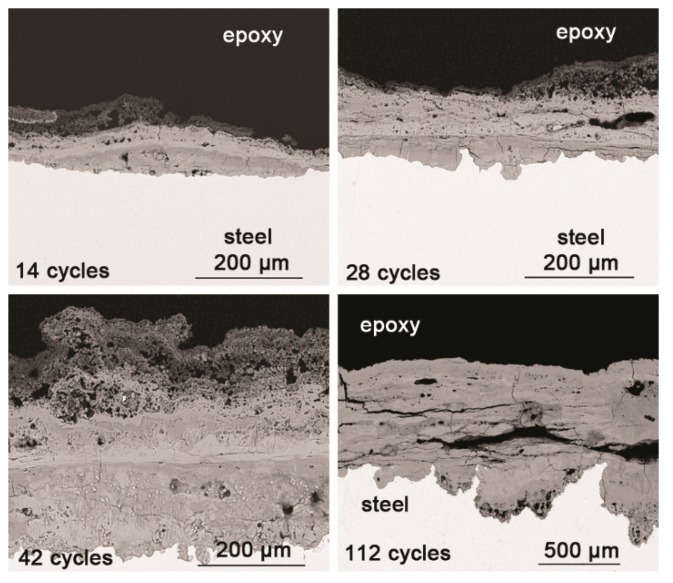
Cross-section SEM micrographs of steel specimens after different numbers of cycles with sufficient fresh salt deposition in each cycle (>116,880 mg/m^2^).

**Figure 7:**
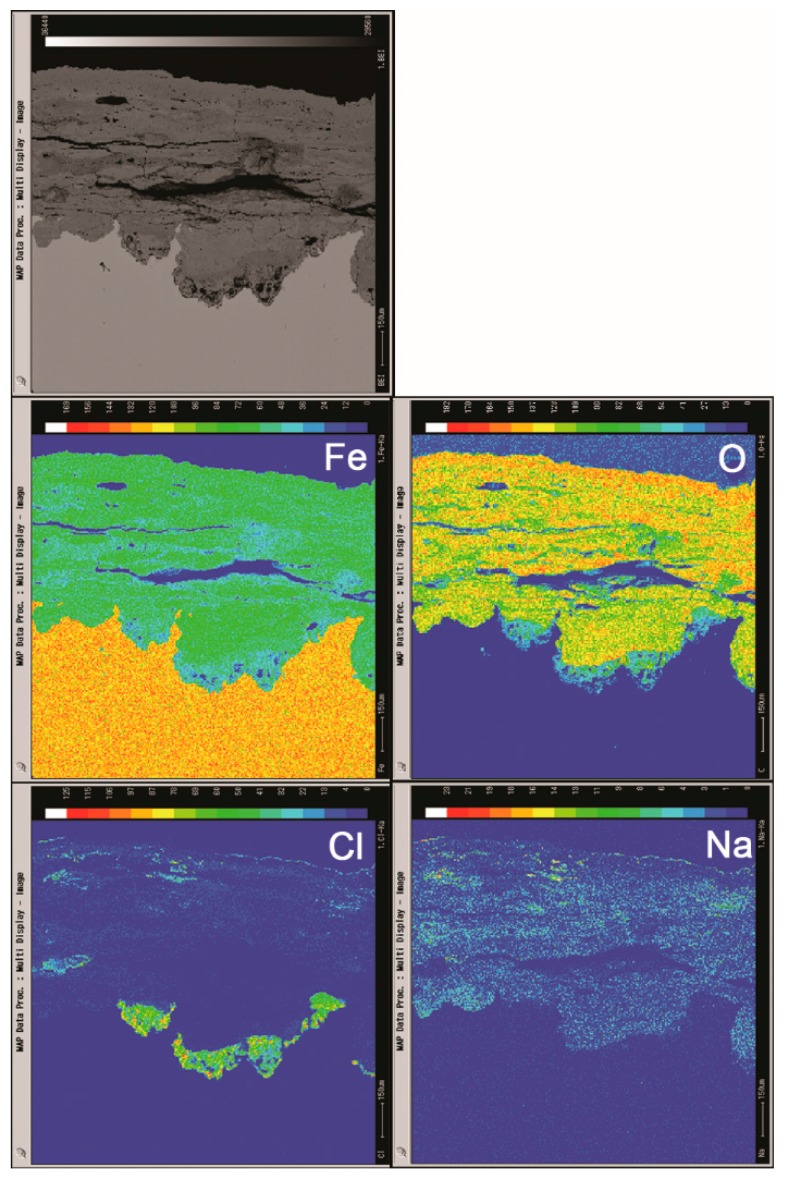
Cross-section observation and EPMA results of rust layers formed on steel specimens after 112 cycles with sufficient fresh salt deposition in each cycle (>116,880 mg/m^2^).

Cross sections of the rust layers formed after different number of cycles were observed by SEM, and the micrographs are shown in Figure 6. The thickness of the layer after 112 cycles was approximately 1 mm. A large number of cracks and pores were present in the rust layer. The EPMA analysis (Figure 7) was carried out to determine if there was an adequate supply of salt in the rust layer. Chlorine was observed to gather at the bottom of the rust layer and existed on the outer layer. Sodium was distributed relatively homogeneously throughout the rust layer except in the chloride-rich region. EPMA confirmed the presence of a substantial amount of chloride in the bottom of rust layer, despite the obstruction of a thicker rust layer.

Based on the results of the experiments under both sets of conditions, the relative amount of akaganeite decreased during long-term atmospheric corrosion, regardless of the salt deposition conditions. The evolution of akaganeite under COND 2 revealed that the proportion of akaganeite declined with prolonged exposure even though there was an adequate amount of chlorine in the inner layer. Therefore, the decrease in the proportion of akaganeite after numerous wet-dry cycles may not be completely due to the lack of chloride, as suggested by previous reports; rather, it may be caused by other reasons.

### 3.2. The Influence of Salt Deposition on the Evolution of Akaganeite

**Figure 8:**
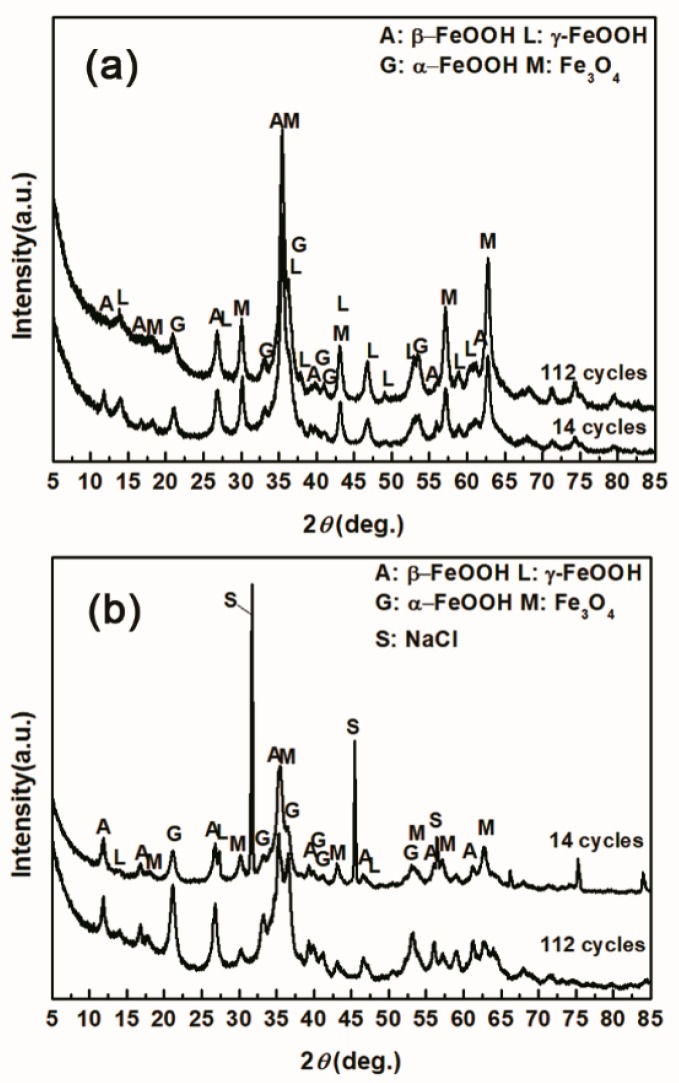
XRD patterns of the rust layer formed on the surfaces of samples after different numbers of cycles with constant salt deposition; (**a**) 1168.8 mg/m^2^; (**b**) 116,880 mg/m^2^.

**Figure 9:**
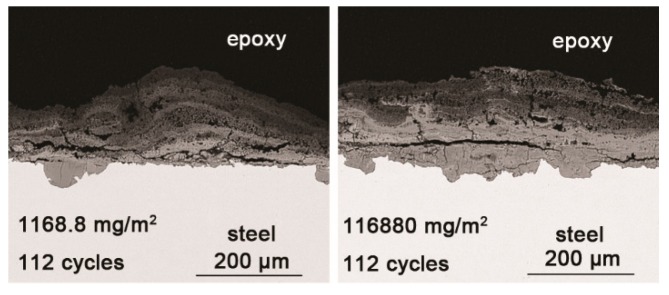
Cross-section SEM micrographs of steel specimens after 112 cycles with constant salt deposition (1168.8 mg/m^2^ and 116,880 mg/m^2^).

To investigate the influence of the amount of salt initially deposited, on the evolution of akaganeite, samples with initial deposits of 1168.8 mg/m^2^ and 116,880 mg/m^2^ of salt were subjected to wet-dry cyclic tests under COND 1. XRD spectra of the rust layers, formed after 14 and 112 cycles, with different initial salt deposits, are displayed in Figure 8. After 14 cycles, lepidocrocite, goethite, magnetite and akaganeite were detected regardless of the salt deposition. However, after 112 cycles, akaganeite was not observed in the sample initially treated with 1168.8 mg/m^2^ of salt. The compositions, determined by MAUD, of the rust layers formed after different numbers cycles are listed in Table 3. The results confirmed that the relative amount of akaganeite decreased after a great deal of wet-dry cycles. A cross-section of a rust layer formed after 112 cycles is presented in Figure 9. The thickness of the rust layer after 112 cycles with an initial deposit of 116,880 mg/m^2^ of salt was approximately 200 μm.

**Figure 10:**
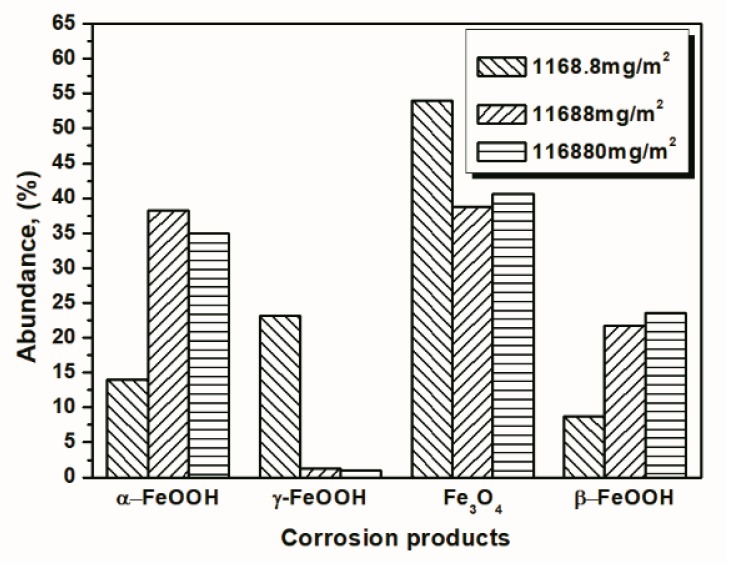
Composition in wt % of the rust layer formed on the surfaces of samples after 14 cycles with constant salt deposition; the data are shown in Table 1, Table 2 and Table 3.

**Figure 11:**
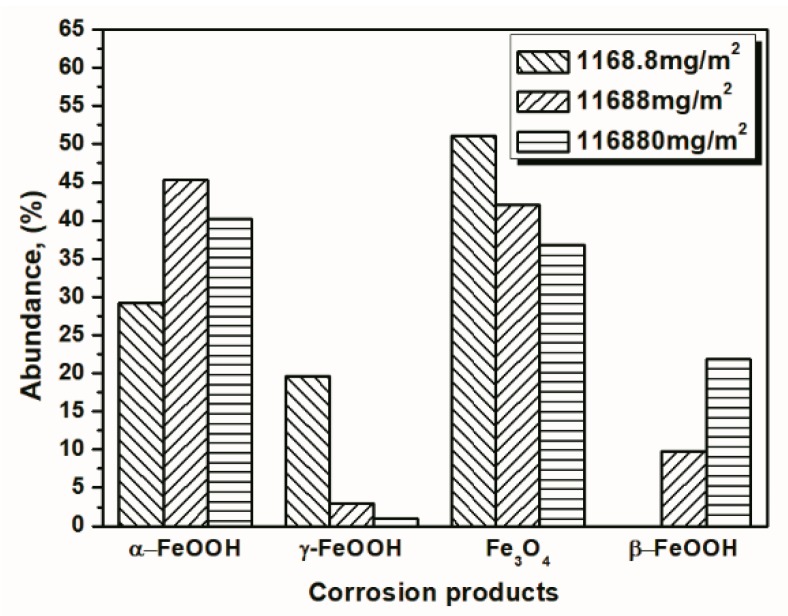
Composition in wt % of the rust layer formed on the surface of samples after 112 cycles with constant salt deposition; the data are shown in Table 1, Table 2 and Table 3.

To better visualize the relative fraction of each phase from different salt deposition conditions, histograms of the relative amounts of each phase after 14 and 112 cycles were prepared by combining the results the 11,688 mg/m^2^ samples and the data shown in Figure 10 and Figure 11. As shown in the figures, the proportion of akaganeite after repeated wet-dry cycles was correlated with the initial salt deposition. Additionally, akaganeite may eventually be depleted when the initial salt deposition is relatively low.

### 3.3. The Process of the Evolution of Akaganeite in the Rust Layer

**Figure 12:**
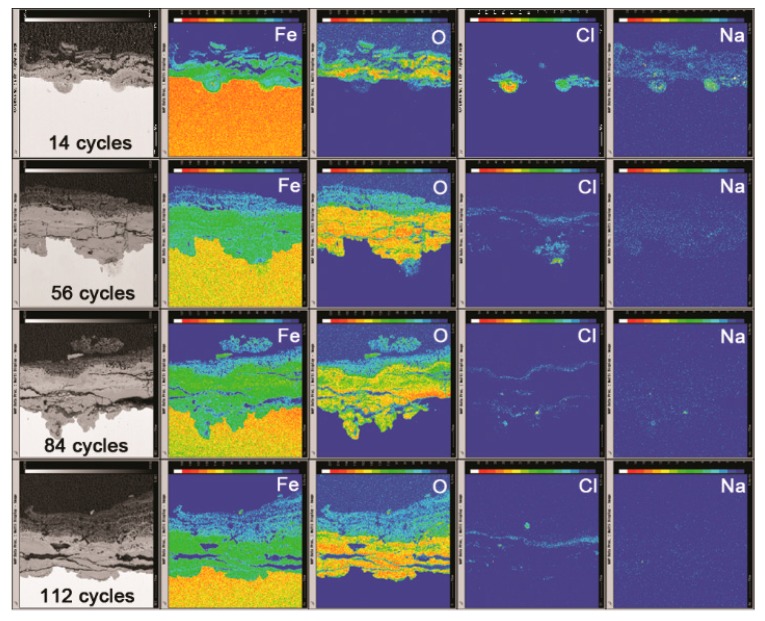
Cross-section observations and electron probe microanalysis (EPMA) results of rust layers formed on steel specimens after different numbers of cycles with constant salt deposition (11,688 mg/m^2^).

The mechanism of the evolution of akaganeite in the rust layer with a constant salt deposition of 11,688 mg/m^2^ was investigated by monitoring the distribution of Fe, O, Cl and Na in the rust layer though EPMA. Figure 12 displays the distribution of each element in the rust layers, formed after 14, 56, 84 and 112 cycles, with an initial salt deposition of 11,688 mg/m^2^. After 14 cycles, chloride was concentrated at the bottom of the thick part of the rust layer, and sodium was distributed relatively uniformly throughout the rust layer. Additionally, sodium was also detected in the region where chloride had aggregated, which indicated that salt is present at the bottom of the thick part of the rust layer. After 56 cycles, chloride was distributed at the top and bottom of the compact rust layer, but it was only present at the top of the compact layer after 112 cycles. According to the XRD analysis, NaCl can be detected after 14 cycles, but is absent after 28 cycles. 

The simulated experiments under COND 1 provided an excellent opportunity to clarify the migration of deposited salt during the wet-dry cycles, since no salt was washed away or deposited after the initial deposition. The mechanisms of the migration of salt and chloride may be that during the initial cycles (cycles 1–14), crystalline salt existed in the rust layer, and in the wet stage of the wet-dry cycles, salt dissolved in the electrolyte to generate Na^+^ and Cl^−^. Underneath the rust layer, the cathodic area and the anodic area separated [25]. The oxidation of iron to Fe^2+^ at the anode must occur on the substrate. To maintain charge neutrality, the electric field forces positive ions in the electrolyte, such as Na^+^, to migrate to the cathodic area and the negative ions, such as Cl^−^, to assemble in the anodic area. As more iron corroded and generated Fe^2+^, more Cl^−^ migrated to the anodic area. Therefore, the amount of separation between Na^+^ and Cl^−^ correlated to the extent of corrosion. The Na^+^ and Cl^−^ that did not separate and migrate to maintain charge neutrality will recrystallize into NaCl once the concentration exceeds the saturation point. However, the remaining Na^+^ and Cl^−^ are dissolved in the electrolyte and move downward as the electrolyte level falls. Consequently, as the electrolyte evaporates, salts crystalize at the bottom of the rust layer. According to the distribution of Na in the rust layer after 14 cycles, Na was fixed in corrosion products and became insoluble, so it could not migrate to the bottom of the rust layer as the electrolyte levels fell.

**Figure 13:**
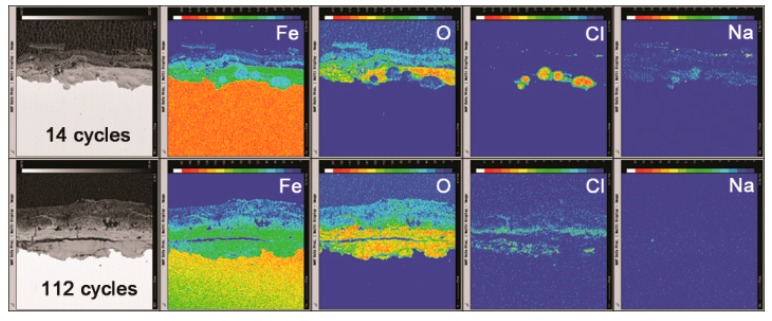
Cross-section observations and EPMA results of the rust layer formed on steel specimens after different numbers of cycles with constant salt deposition (116,880 mg/m^2^).

After several wet-dry cycles (28–112 cycles), all the Na^+^ and Cl^−^ were separated, and NaCl crystals were no longer detected by XRD in the rust layer. After the rust layer was wet with deionized water, the concentration of chloride in the electrolyte on top of the rust layer approached zero, and diffusion caused chloride to migrate from the aggregation region to the outer layer. At the cathode, oxygen was reduced to generate OH^−^. Magnetite was the only conductive corrosion phase and existed mainly in the dense rust layer and rarely in outer rust layer [26]. Therefore, oxygen accepts electrons via the dense rust layer, and it generates OH^−^ on the top of the dense rust layer through reduction. Fe^2+^ and chloride, which migrate based on diffusion, will react with the OH^−^ that is abundant and fixed in this region. However, the fixed chloride was insoluble. Free chloride would move downward toward the bottom of the rust layer as the electrolyte levels fall during drying. Thus, chloride was not detected in the diffusion path. After a number of wet-dry cycles, chloride migrated from the bottom of the rust layer to the top of the compact rust layer and became trapped in corrosion products. This phenomenon, shown in Figure 13, caused the distribution of elements in the rust layer of the 116,880 mg/m^2^ to change. In this case, some chloride still existed in the bottom of the rust layer after 112 cycles because the initial amount of salt deposited was so high.

According to the migration of chloride during repeated wet-dry cycles under COND 1, chloride ions gradually become immobile in the corrosion products. Consequently, the number of soluble chloride ions in the rust layer available to form akaganeite decreased over the course of the corrosion process. Finally, the relative amount of akaganeite declined as corrosion proceeded.

Compared to the corrosion process with constant salt deposition (COND 1), in the condition with sufficient fresh salt deposition in each cycle (COND 2), the electrolyte added in each cycle contained a high concentration of chloride ions. The migration of chloride from the bottom of the rust layer outward due to diffusion was avoided. Under COND 2, the chloride was distributed in the bottom of the rust layer even after numerous wet-dry cycles. The aggregation of Cl in the anodic area made the corrosion conditions worse and accelerated the corrosion rate. Due to this, the thickness of the rust layer formed under COND 1, with 116,880 mg/m^2^ of salt, was far less than the rust layer formed under COND 2.

Particular emphasis can be placed on the comparison between the compositions of the rust layers formed on samples under COND 1 with 116,880 mg/m^2^ and those formed under COND 2. In the case of COND 2, the amount of salt deposition in the rust layer was larger than that of COND 1, at 116,880 mg/m^2^. However, the relative amount of akaganeite was noticeably less in the case of COND 2 than it was in the case of COND 1, after 112 cycles. This phenomenon suggests that the evolution of akaganeite was not solely determined by salt deposition. Although a sufficient amount of fresh chloride was supplied in each cycle, the fraction of akaganeite was reduced by the coverage of a thicker rust layer. 

Akaganeite is a product of the transformation of green rust, which is an Fe(I)–Fe(II) hydroxyl salt [9,10]. Thus, the formation of akaganeite can be divided into two steps, namely, the formation and the oxidation of green rust. For the first step, Fe^2+^ and OH^−^ are required. For the second stage, oxygen is necessary to oxidize the green rust. The Fe^2+^ starting material was obtained by the corrosion of the substrate, which occurs under the rust layer. However, oxygen and OH^−^ are generated by the cathodic reduction of oxygen originating from the atmosphere above the rust layer. Therefore, thicker rust layers hinder the migration of the required substances within the rust layer, which leads to a lower relative amount of akaganeite. The exfoliation of the rust layer under outdoor conditions may also impact the evolution of akaganeite. 

The mechanism of the evolution of akaganeite also influences the location of the akaganeite within the rust layer, which is still being studied [11,18]. Some researchers have found that akaganeite is typically distributed in the surface region of the rust layer, probably due to the reaction between iron ions produced by corrosion and Cl^−^ ions deposited by the atmosphere [27,28]. However, other researchers [18,29,30,31] have found that akaganeite forms in the inner part of the corrosion layer, near the metal–oxide interface. For example, Nomura [32] suggested that an oxygen deficit and the presence of a high concentration of Cl^−^ are fundamental factors for akaganeite formation, and as a result, akaganeite forms in the interior of the rust layer at the steel/rust interface. Other studies have shown that akaganeite is distributed in both the outer and inner layers [33,34]. Heidis Cano found that akaganeite is generally found with lepidocrocite in the outermost corrosion layer and also existed in the inner rust layer associated with the existence of cracks that connect the outer and inner zones [35]. The results of this work showed that the distribution of chloride and salt varied as the number of wet-dry cycles changed and that their distribution was influenced by the mode of salt deposition, the washing of the precipitate, and other factors. Thus, the location of akaganeite in the rust layer may depend on the corrosion process, which requires further study.

## 4. Conclusions

During long-term atmospheric corrosion, the relative amount of akaganeite tends to decrease, even with abundant fresh chloride deposition. When there was a constant amount of salt deposited, the decline in the relative akaganeite content could be attributed to the decrease in the number of soluble chloride ions available. When a sufficient amount of fresh salt was deposited in each cycle, the main reason for the decline in the relative akaganeite content was coverage by an increasingly thick rust layer that hindered the movement of ions. The different chloride migration behaviors under different salt deposition conditions made us speculate that there is not just one location for akaganeite formation within the rust layer, and it varies depending on the corrosion conditions.

## Figures and Tables

**Table 1:** Composition in wt % of the rust layer formed on the surfaces of the samples after different numbers of cycles with constant salt deposition (11,688 mg/m^2^).

Numbers of Cycles	α-FeOOH	γ-FeOOH	Fe_3_O_4_	β-FeOOH
14 cycles	38.25	1.22	38.82	21.70
28 cycles	43.77	3.34	34.50	13.28
56 cycles	53.14	3.58	32.18	11.10
84 cycles	47.11	6.60	36.23	10.06
112 cycles	45.29	2.90	42.02	9.78

**Table 2:** Composition in wt % of the rust layer formed on the surfaces of the samples after different numbers of cycles with sufficient fresh salt deposition in each cycle (>116,880 mg/m^2^).

Numbers of Cycles	α-FeOOH	γ-FeOOH	Fe_3_O_4_	β-FeOOH
14 cycles	24.49	0.94	54.77	19.80
28 cycles	27.69	0.73	45.02	26.56
42 cycles	30.28	1.43	38.76	29.52
112 cycles	41.66	6.73	37.85	13.75

**Table 3:** Composition in wt % of the rust layer formed on the surfaces of samples after different numbers of cycles with constant salt deposition (1168.8 mg/m^2^ and 116,880 mg/m^2^).

Numbers of Cycles (Salt Deposition)	α-FeOOH	γ-FeOOH	Fe_3_O_4_	β-FeOOH
14 cycles (1168.8 mg/m^2^)	14.04	23.17	54.06	8.73
112 cycles (1168.8 mg/m^2^)	29.20	19.69	51.11	0
14 cycles (116,880 mg/m^2^)	34.96	0.94	40.59	23.51
112 cycles (116,880 mg/m^2^)	40.28	1.04	36.77	21.91
